# DNMT inhibition epigenetically restores the cGAS-STING pathway and activates RIG-I/MDA5-MAVS to enhance antitumor immunity

**DOI:** 10.1038/s41401-025-01639-y

**Published:** 2025-08-19

**Authors:** Yao Tu, Qing-yun Zhu, Wen-jun Huang, Sha Feng, Yu-ling Tan, Lu-lu Li, Xin-tong Xie, Qin-yuan Li, Shou-hui Huang, Cheng-zhou Mao, Bi-zhu Chu, Yu-yang Jiang

**Affiliations:** 1 Guangdong Provincial Key Laboratory of Chinese Medicine Ingredients and Gut Microbiomics, School of Pharmacy, Shenzhen University Medical School, Shenzhen University, Shenzhen, 518060 China; 2 The First Affiliated Hospital, Cancer Research Institute, Hengyang Medical School, University of South China, Hengyang, 421001 China; 3 State Key Laboratory of Chemical Oncogenomics, Tsinghua Shenzhen International Graduate School, Shenzhen, 518055 China; 4 Department of Breast Surgical Oncology, Shenzhen Center, Cancer Hospital Chinese Academy of Medical Sciences, Shenzhen, 518116 China; 5 Department of Anatomy and Histology, Shenzhen University Medical School, Shenzhen University, Shenzhen, 518060 China; 6 School of Pharmaceutical Sciences, Tsinghua University, Beijing, 100084 China

**Keywords:** antitumor immunity, immunotherapy response predictor, DNMT inhibitors, cGAS-STING, RIG-I/MDA5-MAVS, epigenetic modulation

## Abstract

The cGAS-STING cytosolic DNA-sensing pathway is a key mediator of the innate immune response and plays a crucial role in antitumor immunity. The expression of cGAS and STING is often suppressed in tumor cells, and reduced expression is associated with poor prognosis and inferior response to immunotherapy. In this study we systematically investigated the expression pattern of cGAS-STING pathway in tumors and its correlation with immunotherapy response. We showed that the expression of cGAS and STING was significantly decreased or undetectable in most breast cancer and murine tumor cell lines, while high cGAS and STING expression was associated with increased T cell infiltration, elevated PD-L1 and PD-1 levels, improved immunotherapy response and prolonged survival. In cGAS-STING–deficient MDA-MB-453 cells, DNMT inhibitor decitabine (DAC, 0.05−1 μM) dose-dependently restored the impaired pathway by reversing DNA methylation–mediated silencing. Furthermore, DAC combined with a chemotherapeutic agent cisplatin significantly enhanced the antitumor effect in MDA-MB-453 and MDA-MB-231 cells by activating the cGAS-STING pathway through cytoplasmic DNA accumulation. In addition, DNMT inhibition elevated intracellular dsRNA levels and activated the RIG-I/MDA5-MAVS pathway. These results suggest that DNMT inhibitors can epigenetically reprogram the cGAS-STING pathway, activate the RIG-I/MDA5-MAVS pathway, and in combination with chemotherapeutic agents, synergistically promote antitumor immunity. Together, this study identifies cGAS-STING as a potential predictor of immunotherapy response and highlights a novel therapeutic strategy for restoring innate immune function in cancer.

**Figure:**
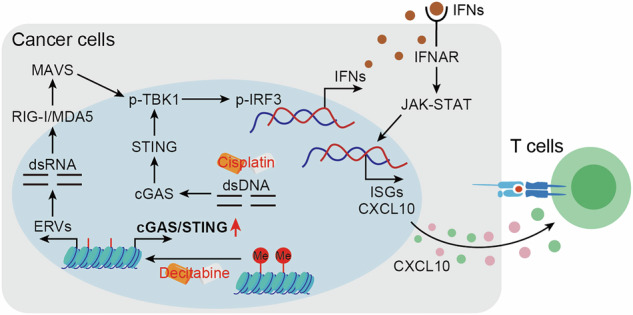
Loss of cGAS-STING signaling in tumors impairs antitumor immunity and correlates with poor immunotherapy response. DNMT inhibition restores cGAS-STING pathway and concurrently activates the RIG-I/MDA5-MAVS signaling, synergistically enhancing immune infiltration and antitumor efficacy.

Loss of cGAS-STING signaling in tumors impairs antitumor immunity and correlates with poor immunotherapy response. DNMT inhibition restores cGAS-STING pathway and concurrently activates the RIG-I/MDA5-MAVS signaling, synergistically enhancing immune infiltration and antitumor efficacy.

## Introduction

Recent advances in cancer treatment have substantially improved patient survival, with immunotherapy emerging as a particularly effective modality [1, 2]. Despite its success in various malignancies, a substantial proportion of patients fail to respond to immunotherapy due to low tumor antigenicity, and impaired immune cell infiltration [3, 4]. Therefore, there is an urgent need to identify reliable biomarkers to predict immunotherapy response and to develop novel strategies to enhance the efficacy of immunotherapy. Tumor-derived interferons (IFNs) can enhance the expression of antigen presentation genes, promote the recruitment of dendritic cells (DCs) and CD8^+^ T cells into the tumor microenvironment through autocrine and paracrine signaling pathway, and thereby enhance the therapeutic efficacy of immunotherapy [5, 6]. Therefore, inducing tumor cell-intrinsic IFNs production represents a potential therapeutic strategy to improve the efficacy of immunotherapy [7, 8]. Recent studies have revealed that chemotherapy and targeted therapies can induce genomic instability, leading to the accumulation of abnormal nucleic acids in the cytoplasm, including double-stranded RNA (dsRNA) [9, 10] and double-stranded DNA (dsDNA) [11]. These aberrant nucleic acids are recognized by specific pattern recognition receptors, such as retinoic acid-inducible gene I (RIG-I)/melanoma differentiation-associated protein 5 (MDA5), and cyclic GMP-AMP synthetase (cGAS), which subsequently activate adaptor proteins like mitochondrial antiviral signaling protein (MAVS) and stimulator of interferon genes (STING), thereby promoting the production of IFNs [8, 12]. In addition, the released IFNs bind to IFN receptors, inducing the expression of interferon-stimulated genes (ISGs) [13]. Therefore, by activating dsRNA and dsDNA signaling pathways, IFNs production can be effectively triggered in the tumor microenvironment, showing promise for tumor immunotherapy.

The cGAS-STING signaling pathway is fundamental to host defense, inducing the expression and secretion of type I IFNs and ISGs to establish an effective natural immune response [14]. In this pathway, cGAS detects aberrant dsDNA in the cytoplasm and catalyzes the synthesis of the secondary messenger 2’3’-cyclic-GMP-AMP (cGAMP). cGAMP binds to the adaptor protein STING (encoded by TMEM173), which recruits TANK-binding kinase 1 (TBK1) to generate a sequential phosphorylation cascade, activating transcription factors such as interferon regulatory factor 3 (IRF3). Phosphorylated IRF3 enters the nucleus, leading to the expression and secretion of type I IFNs and chemokines like CXCL10, which combat pathogenic microorganisms and maintain tissue homeostasis [15, 16]. Accumulating evidence indicates that the cGAS-STING signaling pathway plays a crucial role in antitumor immunity [17–20]. However, the expression of cGAS and STING is often suppressed in tumor cells, and reduced expression is associated with poor prognosis and inferior response to immunotherapy [5, 21–23]. Despite its immunostimulatory potential, the expression of the cGAS-STING pathway in tumors, and its clinical relevance as a predictive biomarker for immunotherapy response have not been systematically investigated. Recent studies suggest that DNA methylation of the cGAS or STING promoter suppresses pathway activity [5, 24–26]. Based on this, we hypothesize that promoter methylation silences cGAS-STING signaling, contributing to immune evasion and tumor progression.

In this study, we found that cGAS-STING pathway is frequently deficient in tumors, while high cGAS and STING expression is associated with increased T cell infiltration, elevated PD-L1 and PD-1 levels, improved immunotherapy response, and prolonged survival. Mechanistic studies show that epigenetic silencing of the cGAS-STING promoter reduces its expression, while DNMT inhibitors restore pathway activity. Combined with the DNA-damaging chemotherapeutic agent cisplatin, DNMT inhibitor decitabine enhances the innate immune response and promote antitumor effects. DNMT inhibition also induces the expression of endogenous retroviruses (ERVs), generating dsRNA that activates the RIG-I/MDA5-MAVS pathway. This dual activation synergistically amplifyed the antitumor immune response and inhibited tumor progression. Collectively, our findings identify cGAS-STING not only as a therapeutic target but also as a predictive biomarker of immunotherapy responsiveness, supporting a novel epigenetic–immune combination strategy for cancer treatment.

## Materials and methods

### Cell lines and cell culture

MDA-MB-453, MDA-MB-231, MDA-MB-436, T-47D, MCF-7, BT-549, Caco-2, A549, MC38, CT26, 4T1 and HEK-293T were kindly provided by Cell Bank, Chinese Academy of Sciences (Shanghai, China). MDA-MB-453, MDA-MB-231, T-47D, MCF-7, BT-549, A549, MC38, CT26, 4T1 and HEK-293T cells were cultured in Dulbecco’s modified Eagle’s medium (Corning, #10-013-CVRC) supplemented with 10% fetal bovine serum (FBS) (Gbico, #10099-141C) and 1% penicillin-streptomycin (Thermo_Gibco™, #15140122). MDA-MB-436 cell lines were cultured in Leibovitz’s L-15 medium (Procell, #PM151010) supplemented with 10% FBS and 1% penicillin-streptomycin. The MDA-MB-361 cell line was cultured in Leibovitz’s L-15 medium supplemented with 20% FBS and 1% penicillin-streptomycin. Caco-2 cells were cultured in Minimum Essential Medium (MEM) (Procell, #PM150410) supplemented with 20% FBS, 1% penicillin-streptomycin, and 1 mM sodium pyruvate. MDA-MB-436, and MDA-MB-361 cell lines were incubated at 37 °C in a humidified atmosphere without CO_2_, while the remaining cell lines were cultured at 37 °C in a humidified environment with 5% CO_2_.

Olaparib (Selleck, #S1060) and Vorinostat (Selleck, #S1047) were purchased from Selleck. The following reagents were obtained from MedChemExpress: Decitabine (#HY-A0004, DAC), Cisplatin (#HY-17394), GSK126 (#HY-13470), KDM5-C70 (#HY-120400), UNC0638 (#HY-15273), JQ-1 (#HY-13030), and SGI-1027 (#HY-13962).

### Immunoblotting analysis

Cells subjected to various treatments were washed with 1× PBS (Smart-lifesciences, #SLB0011), scraped using a cell scraper, and lysed in ice-cold NP-40 buffer (Beyotime, #P0013F) supplemented with protease inhibitors (Roche, #04693132001) and phosphatase inhibitors (Roche, #04906837001). Proteins were separated by electrophoresis using commercially available gels of varying concentrations and transferred onto polyvinylidene fluoride (PVDF) membranes (Merck Millipore, #IPVH00010). The membranes were then incubated overnight at 4 °C with the corresponding primary antibodies, followed by incubation with horseradish peroxidase (HRP)-conjugated secondary antibodies. Actin served as a loading control.

### Quantitative reverse transcription PCR (RT-qPCR) assay

Total RNA was extracted from cells using the Eastep Super Total RNA Extraction Kit (Promega, #LS1040), following the manufacturer’s instructions. RNA concentration was measured using a Nanodrop spectrophotometer (Thermo Fisher, #Nano drop one), and purity was assessed before further processing. Reverse transcription of RNA into cDNA was performed using the HyperScript RT SuperMix kit (APExBiO, #K1074-100). Quantitative PCR (qPCR) was conducted using the Hieff qPCR SYBR Green Master Mix (Low Rox Plus) kit (Yeason, #11202ES08), with reaction components prepared according to the manufacturer’s protocol. qPCR reactions were carried out on a real-time PCR system (Thermo Fisher, #7500 fast), and the resulting data were analyzed. Primer sequences used in this study are listed in Supplementary Table S1.

### Immunofluorescence (IF) assay

Cells were seeded into confocal dishes and incubated at 37 °C with 5% CO_2_. After washing once with 1× PBS, cells were fixed in 500 μL of ice-cold methanol for 10 min at room temperature. Following three washes with 1× PBS, cells were blocked with 500 μL of blocking buffer (1× PBS/5% goat serum (Boster Biological Technology, #AR1009)/0.3% Triton X-100 (Solarbio Biological Technology, #T8200)) for 1 h at room temperature. After blocking, cells were washed three times with 1× PBS and incubated overnight at 4 °C with 300 μL of primary antibody solution prepared in antibody dilution buffer (1× PBS/1% BSA (Sangon Biotech, #A600332)/0.3% Triton X-100) at the following concentrations: STING (1:200), p-STAT1 (1:500), and dsDNA (1:200).

The next day, cells were washed three times with 1× PBS on a shaker (5 min per wash) and incubated with 300 μL of secondary antibody solution prepared in antibody dilution buffer: anti-rabbit (FITC-conjugated Affinipure Goat Anti-Mouse IgG (H + L), 1:200) or anti-mouse (Cy3-conjugated Affinipure Goat Anti-Mouse IgG (H + L), 1:50) for 1 h at room temperature. Following another three washes with 1× PBS (5 min per wash), cells were incubated with 300 μL of DAPI antifluorescence quencher (Yeason, #36308ES20) in the dark for 5 min. Imaging was performed using an ultra-high-resolution laser confocal microscope (Zeiss, #LSM880) at 63× magnification, with at least three fields of view per sample.

### dsDNA or dsRNA stimulation

Cells were seeded in a 6-well plate and transfected using Lipofectamine 3000 (Thermo_Invitrogen™, #L3000015) and Opti-MEM™ I Reduced Serum Medium (Thermo_Gibco™, #31985070), following the manufacturer’s instructions. The transfection mixture contained the specified amounts of Poly (dA:dT) (InvivoGen, #tlrl-patn) or Poly (I:C) (APExBIO, #B5551) as recommended. After a 15-min incubation, the mixture was added to the cells. Following a 4-h transfection period, cell samples were collected for further analysis.

### RNA sequencing (RNA-seq)

After treatment, cells were washed three times with 1× PBS, and 500 μL of TRIzol reagent was added directly to each dish for cell lysis. Total RNA was extracted, and library preparation and sequencing were conducted by BGI Genomics. Bioinformatic analysis of the RNA-seq data was performed using BGI’s multi-omics analysis platform (https://biosys.bgi.com/).

### Animal assays

Female BALB/c wild-type mice, aged 6–8 weeks, were purchased from Guangdong Medical Laboratory Animal Center (Foshan, China). Tumor cells were counted, resuspended to the required concentration, and immediately transferred to the animal facility. Mice were anesthetized, and the fur at the injection sites was shaved. CT26 cells (4 ×10^5^ cells) were injected subcutaneously into the dorsal flank, and 4T1 cells (2 ×10^5^ cells) were injected into the fourth mammary fat pad. One-week post-injection, tumor volumes were measured, and the mice were randomly assigned to four groups for treatment or control solvent administration. Decitabine was dissolved in 1× PBS, and cisplatin was dissolved in a mixture of 50% PEG300 (MedChemExpress, #HY-Y0873) and 50% saline (Beyotime, #ST341-500). Intraperitoneal injections of 100 μL of the drug solution were administered: decitabine every three days and cisplatin every seven days. Tumor dimensions were measured every three days using calipers, and tumor volume was calculated using the formula: Volume = (Length × Width × Width)/2, expressed in cubic millimeters.

All animal procedures were approved by the Institutional Animal Care and Use Committee at the Shenzhen University (IACUC-202300066) and were conducted in strict compliance with the regulations set forth by the committee.

### Immunohistochemical (IHC) staining assay

Tumor tissue sections were deparaffinized, and antigen retrieval was performed using a 2-min heat-induced treatment with EDTA buffer (Maixin Biotech. Co., Ltd, #MVS-0099). Following blocking, sections were incubated overnight at 4 °C with the primary antibody. The next day, sections were incubated with the secondary antibody (Maixin Biotech. Co., Ltd, #KIT-5005), followed by staining with DAB (Maixin Biotech. Co., Ltd, #DAB-1031) chromogenic solution and hematoxylin (Baso, #BA4097). Images were acquired using a pathology slide scanner, and three random fields of view were selected for analysis. The percentage and intensity of positively stained cells were automatically quantified using the IHC Profiler plug-in in ImageJ software.

A commercially available breast cancer tissue array was purchased from Outdo Biotech (HBreD090PG01). This study was approved by the Ethics Committee of Outdo Biotech. Informed consent was obtained from all participants. Expression scores of cGAS and STING, as well as CD8, PD-L1, and PD-1 positivity rates are summarized in Supplementary Table S4. The positivity rates for CD8 and PD-1 were defined as the percentage of positively stained lymphocytes among all nucleated cells within the tumor nests, whereas the PD-L1 positivity rate was defined as the percentage of PD-L1-positive tumor cells among all tumor cells in the tumor nests. All evaluations were independently performed by experienced pathologists.

### Bioinformatics analysis

Kaplan–Meier survival curves for breast and lung cancer patients were generated using the Kaplan–Meier Plotter, stratified by cGAS or STING gene expression levels [27, 28]. Kaplan–Meier analysis of overall and progression-free survival was performed using the Kaplan–Meier Plotter, based on cGAS expression levels in cancer patients treated with immunotherapy [29].

STAR-counts and corresponding clinical data for breast cancer were obtained from The Cancer Genome Atlas (TCGA; https://portal.gdc.cancer.gov/). Raw counts were converted to TPM and log_2_ (TPM + 1) normalized. A total of 1101 breast cancer samples with matched RNA-seq and clinical data were included. Patients were stratified into high- and low-expression groups based on the median cGAS or STING expression. Immune cell infiltration was assessed using the TIMER algorithm in TIMER 2.0 (http://timer.cistrome.org/), and multi-gene correlations were visualized as heatmaps using the pheatmap R package. The GSE103668 dataset, comprising 21 breast cancer patients receiving immunotherapy (14 non-responders and 7 responders), was obtained from GEO. Differential expression analysis was performed using the limma package, followed by GO and KEGG enrichment analyses with ClusterProfiler.

### Statistical analysis

All experiments subjected to statistical analysis were repeated at least three times. Data were presented as the mean ± standard deviation (SD) or standard error of the mean (SEM). Statistical analyses were conducted using GraphPad Prism 8.0 software. An unpaired Student’s *t* test was used for comparisons between two independent groups, while one-way analysis of variance (ANOVA) was applied for multiple group comparisons. A statistically significant difference was defined as *P* < 0.05. Kaplan–Meier survival curves were analyzed using the Mantel–Cox log-rank test. Inclusion and exclusion criteria were pre-determined, and no samples or animals were excluded from the analysis.

## Results

### Impairment of the cGAS-STING signaling pathway in tumor

**Fig. 1:**
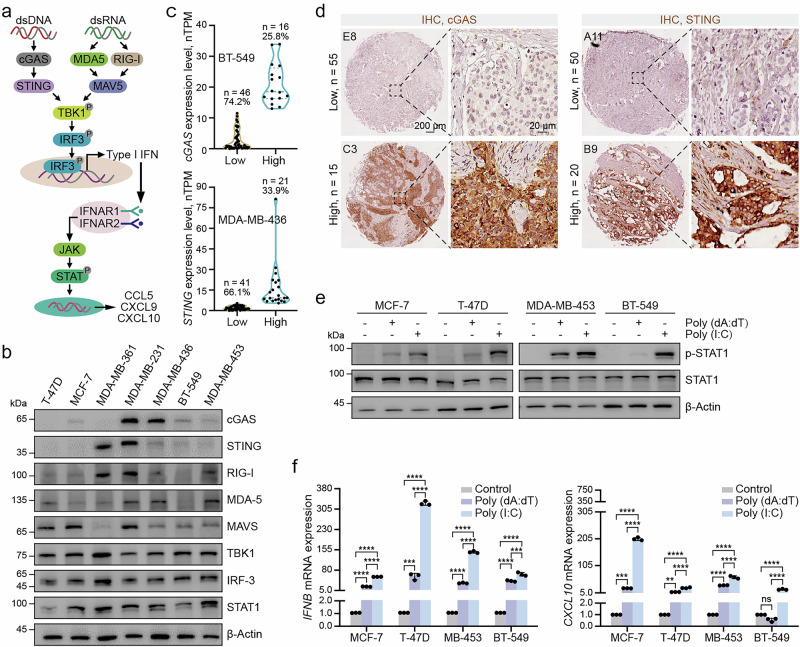
Impairment of the cGAS-STING signaling pathway in tumor cells. **a** Schematic diagrams of the dsDNA and dsRNA sensing pathways. **b** Immunoblot analysis of innate immune signaling pathway-related protein expression levels in different breast cancer cell lines. **c** Bioinformatic analysis of cGAS and STING expression levels in breast cancer cell lines from the HPA database, with BT549 and MDA-MB-436 representing the upper range of expression. **d** Representative IHC staining images showing low and high expression of cGAS and STING in breast cancer tissue arrays. **e** Immunoblot analysis of the indicated proteins in various breast cancer cell lines transfected with 0.5 μg/mL Poly (dA:dT) or Poly (I:C) for 4 h. **f** RT-qPCR analysis of IFNB, and CXCL10 levels in different breast cancer cell lines transfected with 0.5 μg/mL Poly (dA:dT) or Poly (I:C) for 4 h. **P < 0.01, ***P < 0.001, ****P < 0.0001, ns no significant difference.

The cGAS-STING signaling pathway plays a critical role in cellular immune responses, and numerous studies have established its close association with tumorigenesis [30]. We investigated the expression of proteins involved in innate immune pathways, including those that sense dsDNA or dsRNA (Fig. 1a). The results revealed that cGAS and STING expression was low or undetectable in most breast cancer and murine tumor cell lines (Fig. 1b, Supplementary Fig. S1a). The Human Protein Atlas (HPA) data showed low cGAS and STING expression in most breast cancer cell lines, with BT549 and MDA-MB-436 serving as approximate upper expression thresholds (Fig. 1c). IHC analysis of 70 breast cancer samples showed that most tumors had low cGAS and STING expression, based on a 0–3 scoring system where scores of 0–1 were defined as low and 2–3 as high (Fig. 1d, Supplementary Fig. S1b, c). This is consistent with our previous findings and supported by studies reporting cGAS and/or STING dysregulation in colorectal cancer [8, 21], ovarian cancer [31], Merkel cell carcinoma [32], and melanoma [21].

To assess cGAS-STING pathway function, we transfected selected cell lines with dsDNA analog Poly (dA:dT) and dsRNA analog Poly (I:C). Compared to Poly (I:C), Poly (dA:dT) resulted in lower upregulation of phosphorylated TBK1 (p-TBK1) and p-STAT1 (Fig. 1e). RT-qPCR showed low induction of IFNB and the chemokines CXCL9, CXCL10 and CCL5 in these cell lines (Fig. 1f, Supplementary Fig. S1d). These results indicate a potential impairment of the cGAS-STING pathway in tumor cells, which may be linked to tumor immune evasion.

### Intact cGAS-STING signaling correlates with enhanced antitumor immunity and immunotherapy response

**Fig. 2:**
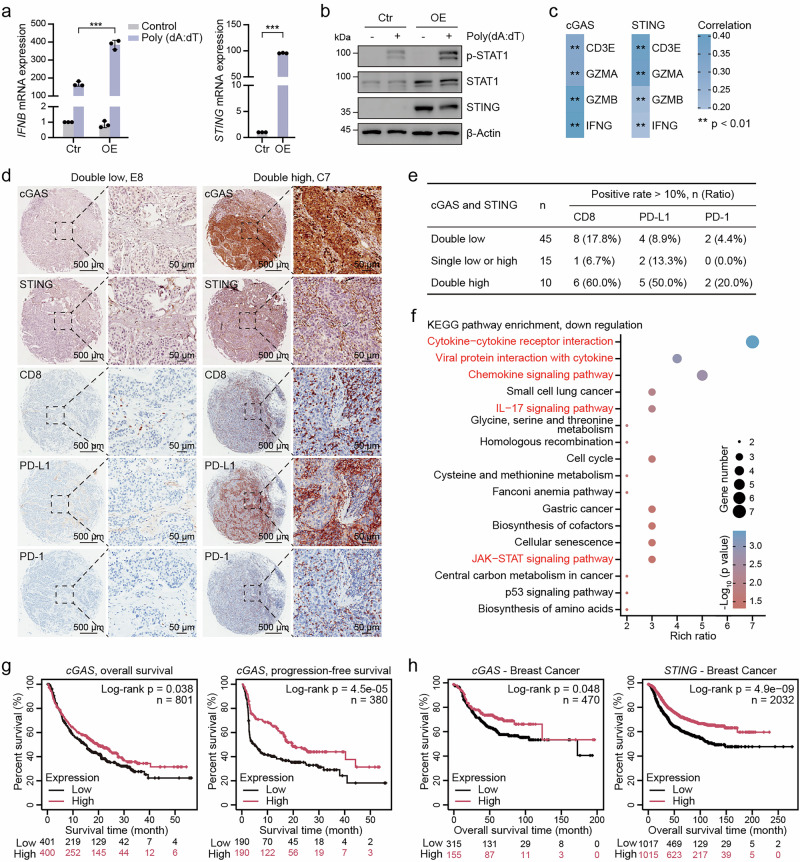
Intact cGAS-STING signaling correlates with enhanced antitumor immunity and immunotherapy response. **a** RT-qPCR analysis of IFNB levels in A549 cells treated with 0.5 μg/mL Poly (dA:dT) following STING overexpression, along with RT-qPCR analysis of STING expression levels in these cells. **b** Immunoblot analysis of the indicated proteins in A549 cells treated with 0.5 μg/mL Poly (dA:dT) following STING overexpression. **c** Correlation analysis of cGAS and STING expression with T cell activation-related genes. **d** Representative IHC staining images of breast cancer tissue arrays showing CD8, PD-L1, and PD-1 expression in samples with either low or high co-expression of cGAS and STING. **e** Proportion of breast cancer patients with >10% positivity for CD8, PD-L1, and PD-1 staining, stratified by cGAS and STING expression levels in tissue arrays. **f** KEGG pathway enrichment analysis of downregulated genes in immunotherapy non-responders from the GSE103668 dataset. **g** Kaplan–Meier overall survival and progression-free survival curves for cGAS expression in patients receiving immunotherapy. **h** Kaplan–Meier overall survival curves of breast cancer patients stratified by cGAS or STING expression. **P < 0.01, ***P < 0.001.

To investigate the functional relevance of STING, we established STING-overexpressing tumor cells. Upon dsDNA stimulation, these cells showed enhanced IFNB expression and increased p-STAT1, indicating restored pathway activity (Fig. 2a, b). Bioinformatic analysis revealed that high cGAS and STING expression was positively associated with increased infiltration of multiple immune cells (Supplementary Fig. S2b), and elevated expression of T cell activation-related genes, including CD3E, GZMA, GZMB, and IFNG (Fig. 2c). Tissue array analysis showed that patients with intact cGAS-STING signaling had higher CD8^+^ T cell infiltration and PD-L1/PD-1 expression, markers predictive of immunotherapy response (Fig. 2d, e, Supplementary Fig. S2a). In addition, KEGG and GO term analyses of immunotherapy-responsive and non-responsive breast cancer patients revealed that innate immunity-related pathways, including cytokine, chemokine, IL-17, and JAK-STAT signaling pathways, were downregulated in non-responsive patients (Fig. 2f, Supplementary Fig. S2c).

Moreover, Kaplan–Meier analysis showed that patients with high cGAS expression had improved overall and progression-free survival following immunotherapy (Fig. 2g). Additionally, low cGAS and STING expression was associated with shorter survival in both breast and lung cancer patients (Fig. 2h, Supplementary Fig. S2d). In conclusion, our results demonstrate that the cGAS-STING pathway play a vital role in tumor immunity, with high expression associated with increased immune cell infiltration and improved patient survival. These findings indicate that cGAS-STING pathway may serve as both a therapeutic target and a predictive biomarker for immunotherapy.

### DNA methyltransferase inhibitors can relieve the repression of cGAS and STING

**Fig. 3:**
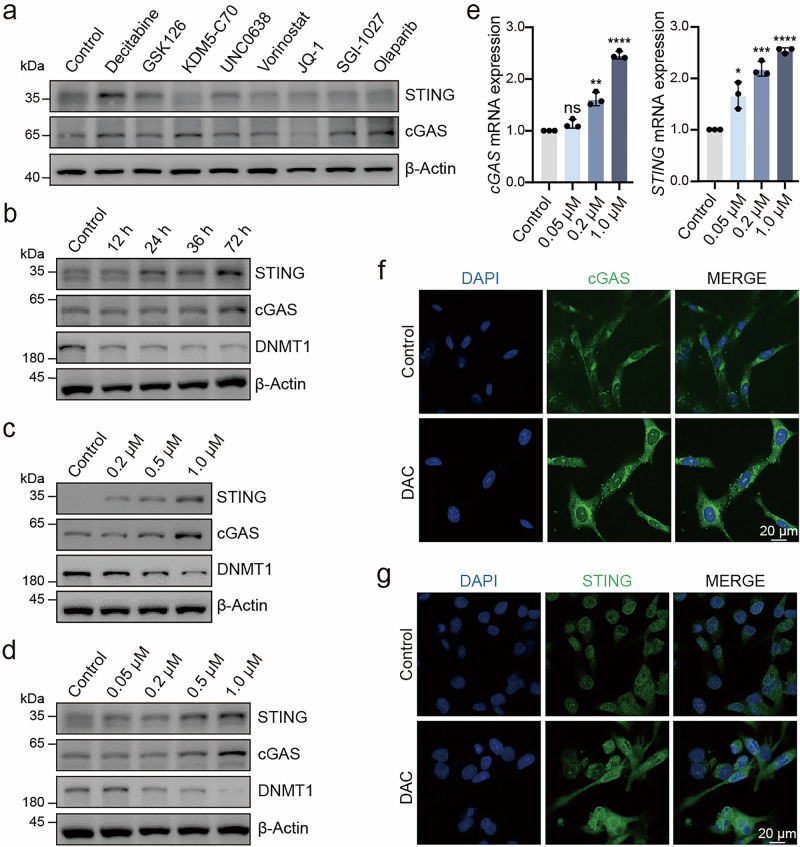
DNMT inhibitor can relieve the repression of cGAS and STING. **a** Immunoblot analysis of the indicated proteins in A549 cells treated with the indicated epigenetic drugs. **b** Immunoblot analysis of the indicated proteins in MDA-MB-453 cells treated with 0.5 μM DAC at different times. Immunoblot analysis of the indicated proteins in A549 (**c**) and MDA-MB-453 (**d**) cells treated with DAC for 72 h. **e** RT-qPCR analysis of cGAS and STING levels in MDA-MB-453 cells treated with DAC for 72 h. IF images of MDA-MB-231 cells stained with DAPI (blue), cGAS (green) (**f**) and STING (green) (**g**) after treatment with 2 μM DAC for 72 h. *P<0.05, **P < 0.01, ***P < 0.001, ****P < 0.0001, ns no significant difference.

Previous studies have demonstrated that cGAS and STING deficiency is frequently caused by loss-of-function mutations or epigenetic silencing of the promoter region. In particular, in human melanoma and glioblastoma cells, the downregulation or loss of gene expression is primarily driven by promoter hypermethylation [22, 33]. Consequently, we employed several epigenetic drugs to explore their potential in restoring cGAS and STING expression. Treatment with the DNA methyltransferase (DNMT) inhibitor decitabine (DAC) significantly activated cGAS and STING expression (Fig. 3a), and prolonged DAC exposure led to further upregulation of cGAS and STING expression (Fig. 3b, Supplementary Fig. S3a). Moreover, increasing DAC concentrations resulted in DNMT1 and DNMT3A inhibition and more pronounced upregulation of cGAS and STING expression (Fig. 3c, d, Supplementary Fig. S3b–e). RT-qPCR and IF assays also confirmed that demethylation treatment effectively restored cGAS and STING expression (Fig. 3e–g, Supplementary Fig. S3f, g). In conclusion, the downregulation or loss of cGAS-STING expression appears to be caused by the DNA methylation, leading to dysfunction of the cGAS-STING signaling pathway. Treatment with DNMT inhibitor to reverse DNA methylation can restore their expression, offering a potential therapeutic strategy for reactivating this critical signaling pathway in cancer cells.

### DNMT inhibitor restores cGAS-STING pathway activation by cytosolic DNA

**Fig. 4:**
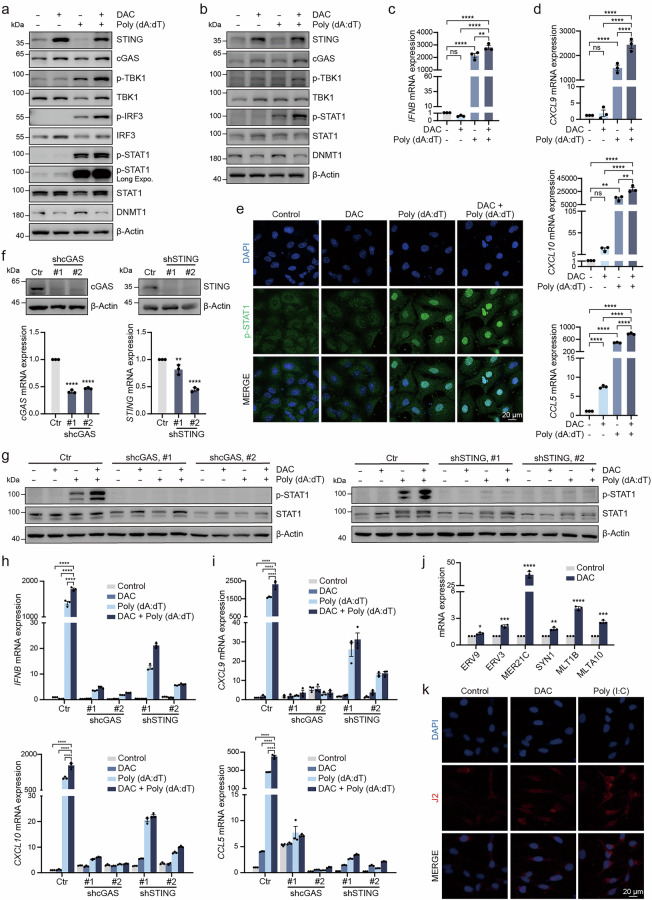
DNMT inhibitor restores cGAS-STING pathway activation by cytosolic DNA. Immunoblot analysis of the indicated proteins in A549 (**a**) and MDA-MB-453 (**b**) cells pretreated with DAC for 72 h and treated with 0.5 μg/mL Poly (dA:dT) for 4 h. RT-qPCR analysis of IFNB (**c**), CXCL9, CXCL10 and CCL5 (**d**) levels in A549 cells pretreated with 1 μM DAC for 72 h and treated with 0.5 μg/mL Poly (dA:dT) for 4 h. **e** IF images of A549 cells stained with DAPI (blue) and p-STAT1 (green) after pretreatment with 1 μM DAC for 72 h and treatment with 0.5 μg/mL Poly (dA:dT) for 4 h. **f** Immunoblot and RT-qPCR analysis of cGAS and STING levels in A549 cells transduced with the indicated shRNA. **g** Immunoblot analysis of the indicated proteins in A549 cells transduced with the indicated shRNA, pretreated with 1 μM DAC for 72 h and treated with 0.5 μg/mL Poly (dA:dT) for 4 h. RT-qPCR analysis of IFNB (**h**) CXCL9, CXCL10 and CCL5 (**i**) levels in A549 cells transduced with the indicated shRNA, pretreated with 1 μM DAC for 72 h and treated with 0.5 μg/mL Poly (dA:dT) for 4 h. **j** RT-qPCR analysis of the ERVs levels after treatment with 1 μM DAC for 72 h. **k** IF images of cells stained with DAPI (blue) and J2 (red) after treatment with 1 μM DAC for 72 h or treatment with 0.5 μg/mL Poly (I:C) for 4 h. *P < 0.05, **P < 0.01, ***P < 0.001, ****P < 0.0001, ns no significant difference.

To evaluate whether DNMT inhibitor restores cGAS-STING functionality, cells were transfected with Poly (dA:dT) to assess the recovery of their expression and activity. Western blot analysis revealed that cells treated with DAC prior to Poly (dA:dT) transfection exhibited a significant increase in the levels of p-TBK1 and p-STAT1 compared to cells transfected with Poly (dA:dT) alone (Fig. 4a, b, Supplementary Fig. S4a–c). RT-qPCR analysis further demonstrate that followed by DAC pre-treatment, Poly (dA:dT) led to higher expression levels of IFNB, CXCL9, CXCL10 and CCL5 (Fig. 4c, d, Supplementary Fig. S4d–f). Additionally, IF assays results show that the p-STAT1 levels and nuclear translocation were significantly higher in the DAC and Poly (dA:dT) co-treated group (Fig. 4e). These results suggest that the recovery of cGAS and STING expression enhances the activation of the downstream TBK1-IRF3 and STAT1 signaling pathways in response to dsDNA.

To further confirm the dependency of cGAS-STING pathway activation on the restoration of cGAS and STING expression and function, we established stable cell lines with cGAS and STING knockdown (Fig. 4f). In these knockdown cell lines, the combined effect of DAC and the Poly (dA:dT) in activating the cGAS-STING pathway was significantly impaired (Fig. 4g), and the expression levels of IFN, and chemokines were also reduced (Fig. 4h, i). These above results suggest that DNMT inhibitor can restore cGAS and STING expression, and that cGAS and STING are essential for maintaining the functionality of these pathways.

Multiple studies have demonstrated that DNMT inhibitor can enhance the expression of ERVs, and induce the formation of dsRNAs, which in turn activate the RIG-I/MDA5-MAVS pathway, triggering a type I IFN response [8, 34, 35]. Our results further show that DAC treatment significantly increases ERV expression (Fig. 4j) and induces the accumulation of intracellular dsRNA (Fig. 4k). These findings suggest that DAC not only restores cGAS-STING pathway but also enhances antitumor activity by activating the RIG-I/MDA5-MAVS pathway, thereby promoting tumor immunogenicity and strengthening antitumor immune responses. Therefore, our study highlights the synergistic activation of innate immune signaling through both the cytoplasmic dsDNA and dsRNA sensing pathways, providing a novel therapeutic strategy to enhance immune responses in cancer treatment.

### Combination of DAC and CDDP enhances cGAS-STING pathway activation

**Fig. 5:**
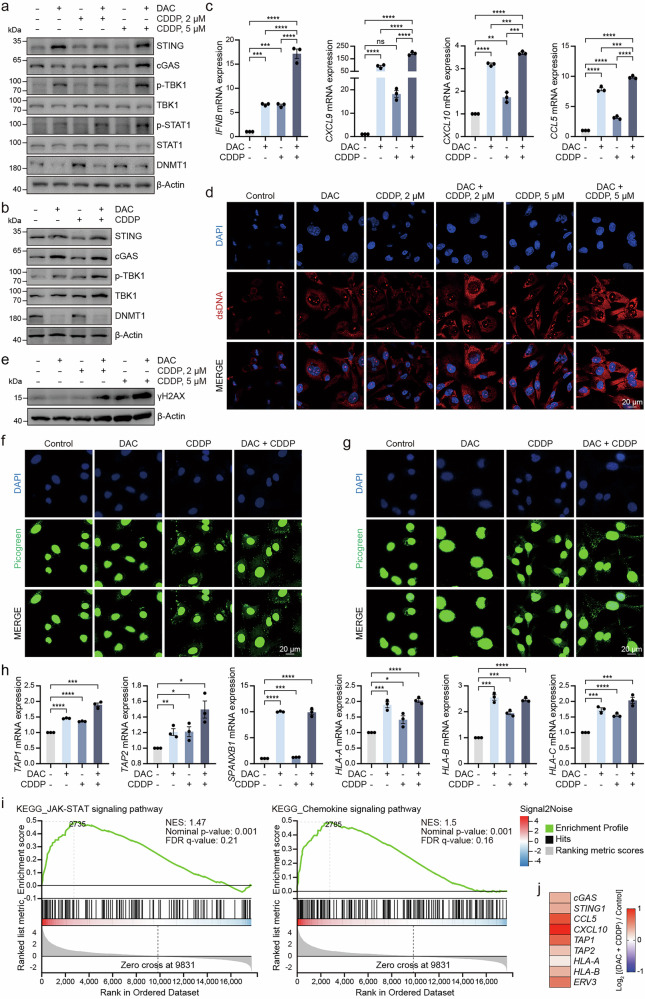
Combination of DAC and CDDP enhances cGAS-STING pathway activation. Immunoblot analysis of the indicated proteins in MDA-MB-453 (**a**) and MDA-MB-231 (**b**) cells pretreated with DAC for 72 h and treated with 2 μM or 5 μM CDDP for 24 h. **c** RT-qPCR analysis of IFNB, CXCL9, CXCL10 and CCL5 levels in MDA-MB-453 cells pretreated with DAC for 72 h and treated with 5 μM CDDP for 24 h. **d** IF images of MDA-MB-231 cells stained with DAPI (blue) and dsDNA (red) after pretreatment with DAC for 72 h and treatment with 2 μM or 5 μM CDDP for 24 h. **e** Immunoblot analysis of the indicated proteins in cells pretreated with DAC for 72 h and treated with CDDP for 24 h. **f** PicoGreen staining of cytosolic dsDNA in A549 cells treated with 1 μM DAC for 72 h and/or 5 μM CDDP for 24 h. **g** PicoGreen staining of cytosolic dsDNA in MDA-MB-231 cells treated with 2 μM DAC for 72 h and/or 5 μM CDDP for 24 h. **h** RT-qPCR analysis of HLA complex gene levels in cells treated with DAC for 72 h. **i** GSEA analysis of RNA-seq data from MDA-MB-231 cells treated with 2 μM DAC for 72 h and 5 μM CDDP for 24 h. **j** Heatmap showing differentially expressed genes associated with the cGAS-STING pathway and immune-related responses in MDA-MB-231 cells treated with DAC and CDDP as described above. *P < 0.05, **P < 0.01, ***P < 0.001, ****P < 0.0001, ns no significant difference.

The above results suggest that treatment with DAC can restore cGAS and STING expression. We hypothesize that combining DAC with a drug that induces dsDNA, thereby activating the cGAS-STING pathway, could enhance antitumor effects by re-establishing the function of cGAS and STING. Cisplatin (CDDP), a widely used chemotherapy drug, activates the cGAS-STING pathway by inducing DNA damage [36]. Therefore, we hypothesize that the combination of DAC and CDDP could restore and activate cGAS-STING pathway. The results showed that the combination of DAC and CDDP led to higher levels of p-TBK1 and p-STAT1 following the restoration of cGAS and STING expression (Fig. 5a, b, Supplementary Fig. S5a, b). RT-qPCR analysis further confirmed the highest levels of IFNB, CXCL9, CXCL10 and CCL5 in the combination group (Fig. 5c, Supplementary Fig. S5c).

Moreover, the cytosolic DNA levels were significantly increased in the combination group, with further increases observed at higher CDDP concentrations (Fig. 5d). To confirm that CDDP activates the cGAS-STING pathway through DNA damage, we measured γH2AX levels and observed a significant increase in γH2AX following CDDP treatment (Fig. 5e, Supplementary Fig. S5d, e). Additionally, PicoGreen staining further confirmed the accumulation of cytosolic dsDNA in both the CDDP and combination treatment groups compared to control, providing direct visual evidence of DNA damage-induced cytosolic DNA accumulation (Fig. 5f, g). We also analyzed the expression of genes related to antigen presentation, particularly those associated with the human leukocyte antigen (HLA) complex. The combination of DAC and CDDP treatment significantly elevated the expression of these genes compared to other groups (Fig. 5h). The above results demonstrate that the combination of DAC and CDDP, by restoring cGAS and STING expression and function, significantly activates the cGAS-STING signaling pathway.

To further investigate the mechanisms of DAC and CDDP combination treatment, we performed RNA-seq on MDA-MB-231 cells. KEGG and GOBP analyses revealed significant enrichment of innate immune-related pathways in the combination group (Supplementary Fig. S5f, g). GSEA showed activation of the JAK-STAT pathway, along with enrichment of chemokine signaling, cytokine-cytokine receptor interactions, and natural killer (NK) cell mediated cytotoxicity (Fig. 5i, Supplementary Fig. S5h). Differential expression analysis further revealed upregulation of cGAS-STING pathway components and chemokines such as CCL5 and CXCL10 (Fig. 5j). These transcriptomic data support that DAC and CDDP synergistically activate innate immune signaling and enhance tumor immunogenicity, potentially improving sensitivity to immunotherapy.

### Combination therapy with DAC and CDDP enhances antitumor immune efficacy

**Fig. 6:**
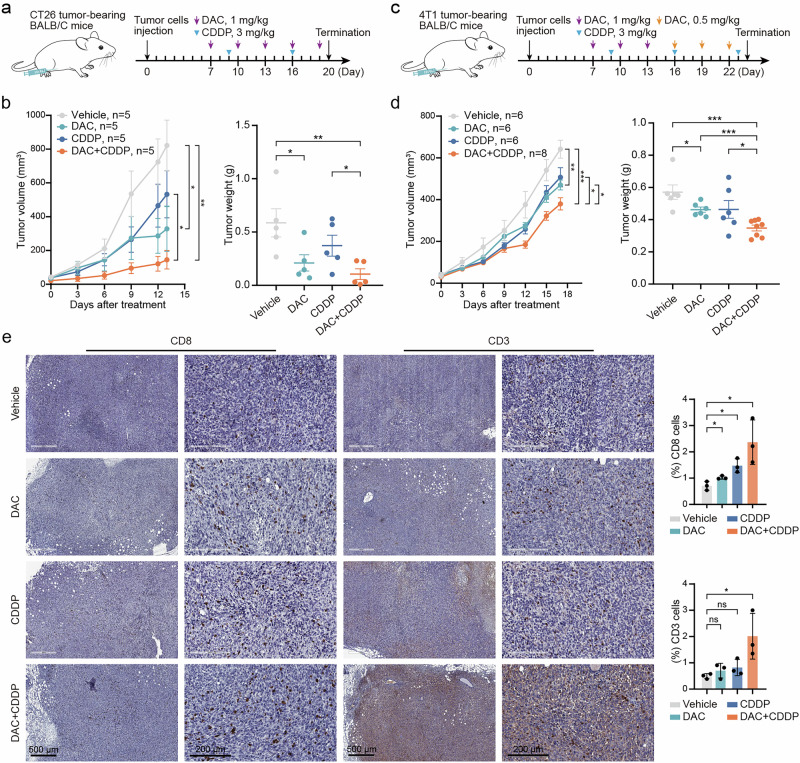
Combination therapy with DAC and CDDP enhances antitumor immune efficacy. **a** Schematic illustrating the administration of DAC and CDDP for tumor treatment in CT26 tumor-bearing BALB/c mice. **b** The tumor growth curve was plotted (*n* = 5), and the tumor weight at the end of the experiments was shown. Error bars represent the mean ± SEM. **c** Schematic illustrating the administration of DAC and CDDP for tumor treatment in 4T1 tumor-bearing BALB/c mice. **d** The tumor growth curve was plotted (*n* ≥ 6), and the tumor weight at the end of the experiments was shown. Error bars represent the mean ± SEM. **e** Representative IHC images of CD3 or CD8 (left) and quantitative analysis (right) from 4T1 tumors. Significance was determined by unpaired Student’s *t* test. *P < 0.05, **P < 0.01; ***P < 0.001, ns no significant difference.

We established subcutaneous CT26 and orthotopic 4T1 tumor models to evaluate the antitumor effects of DAC and CDDP in vivo. Tumor inoculation and treatment administration were performed as shown in Fig. 6a, c. We found that the combination of DAC and CDDP significantly inhibited tumor growth (Fig. 6b, d, Supplementary Fig. S6a, b). Moreover, no weight loss or other signs of toxicity were observed in the mice treated with DAC and CDDP (Supplementary Fig. S6c, d). Mechanistically, the combination treatment significantly restores cGAS and STING expression in vivo, along with elevated γH2AX levels (Supplementary Fig. S6e). Additionally, the combination treatment increased the infiltration of CD3^+^ and CD8^+^ T cells in 4T1 tumors compared to the control and monotherapy groups (Fig. 6e). These results indicate that the combination therapy significantly restores and activates the cGAS-STING pathway, inducing an immune response against the tumor.

## Discussion

The cGAS-STING pathway plays a key role in pathogen detection and the activation of host defenses, while also significantly contributing to antitumor immune responses [37, 38]. The activated cGAS-STING pathway secretes IFNs and chemokines, serving as a crucial bridge between innate and adaptive immunity, facilitating DC maturation, enhancing antigen presentation, and activating CD8^+^ T cells to recognize and eliminate tumor cells. Additionally, it recruits other immune cells, such as NK cells, further strengthening the immune attack against tumors [5–7]. However, cancer cells may evade immune surveillance by silencing cytosolic DNA sensing pathways, leading to reduced or absent expression of cGAS and STING [5, 8, 21, 24]. Previous studies suggest that epigenetic silencing of the cGAS or STING gene promoter regions may contribute to the loss of expression of these proteins [5, 25, 33]. Our studies confirm the impairment of the cGAS-STING signaling pathway in various cancers and identifies DNA methylation-induced silencing of the cGAS or STING genes as a potential cause of this damage. After using the DNA methylation inhibitor DAC, we observed a significant restoration of cGAS or STING expression and function, thereby rescuing the impaired signaling pathway. In cancers where cGAS or STING expression is silenced, reactivating this pathway may be a direct approach to restoring tumor immunogenicity or enhancing sensitivity to immune checkpoint blockade therapy.

Given the crucial role of cGAS-STING signaling in antitumor responses, there has been significant interest in developing synthetic STING agonists. Currently, most STING agonists are synthetic analogs of 2’3’-cGAMP, inspired by its ability to activate STING and trigger downstream IFNs signaling. These agonists include cyclic dinucleotides (CDNs) based on the 2’3’-cGAMP structure, as well as non-CDN molecules. However, synthetic STING agonists often struggle to penetrate cancer cell membranes [39, 40]. We adopted an alternative strategy by increasing the production of endogenous dsDNA within cells to activate the cGAS-STING pathway. Cisplatin, a commonly used chemotherapy drug, can activate this pathway by inducing DNA damage, which releases endogenous dsDNA. By using the DNMT inhibitor to remove the silencing of cGAS or STING, we restored the damaged pathway and significantly enhanced cisplatin-induced cGAS-STING responses. This led to increased secretion of IFNs and various effector chemokines, and MHC molecules, which synergistically boosted antitumor immunity and improved patient sensitivity to cisplatin. Moreover, DAC also can enhance the expression of ERVs and induce the formation of dsRNA, thereby activating the dsRNA-sensing pathway and leading to an IFNs response [8, 34, 35]. Moreover, DNMT inhibitors not only modulate innate immunity in tumor cells but also reprogram immune cells, including T cells and CAR-T cells, toward NK-like phenotypes with enhanced antitumor activity [41]. Thus, comprehensive evaluation of the effects of DNMT inhibition on the tumor microenvironment may reveal broader immunomodulatory effects and further improve therapeutic outcomes.

While immunotherapy has revolutionized cancer treatment, the lack of robust and broadly applicable biomarkers for predicting therapeutic response remains a major clinical challenge. Conventional markers such as PD-L1 expression and tumor mutational burden (TMB) are limited in their ability to reflect the complexity of the tumor immune microenvironment. Our study demonstrates that cGAS-STING pathway is frequently deficient in tumors, and its low expression is associated with poor immune infiltration, reduced immunotherapy responsiveness, and worse patient survival, highlighting its potential as a functional biomarker of tumor immunogenicity and a potential predictor of immunotherapy response. Recent clinical and translational studies have further supported this notion. In colorectal cancer, patients with high cGAS-STING expression exhibit better response rates and longer survival following immune checkpoint blockade [42, 43]. Moreover, responders to immune checkpoint inhibitors patients exhibit stronger IFN responses and enrichment of STING activation [44]. These observations collectively suggest that the integrity of the cGAS-STING pathway may be critical for effective antitumor immunity and clinical benefit from immunotherapy. Further in-depth investigation into the role of cGAS-STING pathway in regulating immunotherapy outcomes could not only improve our understanding of resistance mechanisms but also refine patient stratification and guide more effective use of immunotherapeutic strategies.

## Supplementary information

Supplementary Information

Figure S1

Figure S2

Figure S3

Figure S4

Figure S5

Figure S6
