# Supplementary material for: DNMT inhibition epigenetically restores the cGAS-STING pathway and activates RIG-I/MDA5-MAVS to enhance antitumor immunity
Source: Acta Pharmacol Sin. 2025 Aug 19;47(1):197–208. doi: 10.1038/s41401-025-01639-y (PMC12764874; doi:10.1038/s41401-025-01639-y)
Supplement: Supplementary file 1 — Supplementary Information [file 41401_2025_1639_MOESM1_ESM.docx]

# **Supporting Information**

**DNMT inhibition epigenetically restores the cGAS-STING pathway and activates RIG-I/MDA5-MAVS to enhance antitumor immunity**

# **Fig. S1 to S6.**

**Fig. S1.** (a) Immunoblot analysis of the indicated proteins in various cell lines. (b) IHC staining images of cGAS and STING in a breast cancer tissue array. Dashed boxes indicate adjacent non-tumor regions in the corresponding HE-stained sections. (c) Quantification of breast cancer tissue array samples stratified by cGAS and STING expression scores. (d) RT-qPCR analysis of CCL5, and CXCL9 levels in different breast cancer cell lines transfected with 0.5 μg/mL Poly (dA:dT) or Poly (I:C) for 4 h. ** *P* < 0.01, *** *P* < 0.001, **** *P* < 0.0001, ns: no significant difference.

**Fig. S2.** (a) IHC staining images of CD8, PD-L1, and PD-1 in a breast cancer tissue array, with data kindly provided by Outdo Biotech. (b) TIMER analysis of the correlation between cGAS and STING expression and immune cell infiltration. (c) GO term enrichment analysis of downregulated genes in immunotherapy non-responders. (d) Kaplan-Meier overall survival curves of lung cancer patients stratified by cGAS or STING expression. **** *P* < 0.0001.

**Fig. S3.** (a) Immunoblot analysis of the indicated proteins in MC38 cells treated with 10 μM DAC at different times. (b) Immunoblot analysis of the indicated proteins in MC38 cells treated with DAC for 72 h. (c, d) Immunoblot analysis of the indicated proteins in Caco-2 (c) and MDA-MB-231 (d) cells treated with DAC for 72 h. (e) Immunoblot analysis of DNMT1, DNMT3A, and DNMT3B protein expression in various tumor cells following treatment with DAC. (f, g) IF images of A549 cells stained with DAPI (blue), cGAS (green) (f) and STING (green) (g) after treating with 1 μM DAC for 72 h.

**Fig. S4.** (a) Immunoblot analysis of the indicated proteins in MDA-MB-231 cells pretreated with 2 μM DAC for 72 h and treated with 0.5 μg/mL poly (dA:dT) for 4 h. (b, c) Immunoblot analysis of the indicated proteins in MC38 (b) and CT26 (c) cells pretreated with DAC for 72 h and treated with 0.5 μg/mL poly (dA:dT) for 4 h. (d) RT-qPCR analysis of cGAS and STING levels in MDA-MB-231 cells pretreated with 2 μM DAC for 72 h and treated with 0.5 μg/mL poly (dA:dT) for 4 h. (e, f) RT-qPCR analysis of IFNB (e), CXCL9, CXCL10 and CCL5 (f) levels in MDA-MB-231 cells pretreated with 2 μM DAC for 72 h and treated with 0.5 μg/mL poly (dA:dT) for 4 h. * *P* < 0.05, ** *P* < 0.01, *** *P* < 0.001, **** *P* < 0.0001, ns: no significant difference.

**Fig. S5.** (a) Immunoblot analysis of the indicated proteins in MC38 cells pretreated with DAC for 72 h and treated with 2 μM or 5 μM CP for 24 h. (b) Immunoblot analysis of the indicated proteins in A549 cells pretreated with 1 μM DAC for 72 h and treated with 5 μM CP for 24 h. (c) RT-qPCR analysis of Ifnb1, Cxcl10 and Ccl5 levels in MC38 cells pretreated with DAC for 72 h and treated with 5 μM CP for 24 h. (d) Immunoblot analysis of the indicated proteins in A549 cells pretreated with 1 μM DAC for 72 h and treated with 5 μM CP for 24 h. (e) Immunoblot analysis of the indicated proteins in MDA-MB-231 cells pretreated with 2 μM DAC for 72 h and treated with 5 μM CP for 24 h. (f) KEGG pathway enrichment analysis based on RNA-seq data from MDA-MB-231 cells treated with 2 μM DAC for 72 h and 5 μM CDDP for 24 h. (g) GOBP enrichment analysis of the same RNA-seq dataset. (h) GSEA showing activation of immune-related pathways following combined DAC and CDDP treatment. ** *P* < 0.01, **** *P* < 0.0001, ns: no significant difference.

**Fig. S6.** (a, b) Photographs of representative CT26 (a) and 4T1 (b) tumors at the end of the experiments. (c, d) The body weight of CT26 (c) and 4T1 (d) tumor-bearing BALB/c mice. (e) CT26 tumor-bearing mouse tissue lysates were analyzed by Western blotting with the indicated antibodies. *n* = 5.

# **Table S1.** Primer sequences.

| Gene | Forward primer (5’-3’) | Reverse primer (5’-3’) |
| --- | --- | --- |
| GAPDH | GGAGCGAGATCCCTCCAAAAT | GGCTGTTGTCATACTTCTCATGG |
| CCL5 | CCAGCAGTCGTCTTTGTCAC | CTCTGGGTTGGCACACACTT |
| CXCL9 | AAGACCTTAAACAATTTGCCCC | TGCTGAATCTGGGTTTAGACAT |
| CXCL10 | GTGGCATTCAAGGAGTACCTC | TGATGGCCTTCGATTCTGGATT |
| IFNB | GCCATCAGTCACTTAAACAGC | GAAACTGAAGATCTCCTAGCCT |
| STING | GGGCTGGCATGGTCATATTA | TACTCAGGTTATCAGGCACC |
| cGAS | TAACCCTGGCTTTGGAATCAAAA | TGGGTACAAGGTAAAATGGCTTT |
| SPANXB1 | TGTGAATCCAACGAGGCCAACG | CCACTAGTATGGTCGAGGACTC |
| HLA-A | TCAGATAGAAAAGGAGGGAGTTACA | ACAAGCTGTGAGGGACACAT |
| HLA-B | CCTGAGATGGGAGCCGTCTT | CTCCGATGACCACAACTGCT |
| HLA-C | GGACAAGAGCAGAGATACACG | CAAGGACAGCTAGGACAACC |
| TAP1 | CTCTGGAAACCCTGTGCGT | GCCCGTAAAGAATGGAATGG |
| TAP2 | GACCTTCCACTAGACCATGAGC | GGAGGATTAAGATTAGTAC  GATGGT |
| ERV9 | TCTTGGAGTCCTCACTCAAACTC | ACTGCTGCAACTACCCTTAAACA |
| ERV3 | AACTAATGCCCCAAGATAATTTCA | TTAAGAACCAGATGCTCTGACTTG |
| MER21C | GGAGCTTCCTGATTGGCAGA | ATGTAGGGTGGCAAGCACTG |
| SYN1 | ATGGAGCCCAAGATGCAG | AGATCGTGGGCTAGCAG |
| MLT1B | TGCCTGTCTCCAAACACAGT | TACGGGCTGAGCTTGAGTTG |
| MLTA10 | TCTCACAATCCTGGAGGCTG | GACCAAGAAGCAAGCCCTCA |
| mCcl5 | TGCTGCTTTGCCTACCTC | CTTGAACCCACTTCTTCTCT |
| mCxcl10 | TGAATCCGGAATCTAAGACCATCAA | AGGACTAGCCATCCACTGGGTAAAG |
| mIfnb1 | AGCTCCAAGAAAGGACGAACA | GCCCTGTAGGTGAGGTTGAT |
| mActin | GGCTGTATTCCCCTCCATCG | CCAGTTGGTAACAATGCCATGT |

# **Table S2.** shRNA sequences.

| Gene | 5’-3’ |
| --- | --- |
| shcGAS, #1F | CCGGGATGCTGTCAAAGTTTAGGAACTCGAGTTCCTAAACTTTGACAGCATCTTTTTG |
| shcGAS, #1R | AATTCAAAAAGATGCTGTCAAAGTTTAGGAACTCGAGTTCCTAAACTTTGACAGCATC |
| shcGAS, #2F | CCGGCGTGAAGATTTCTGCACCTAACTCGAGTTAGGTGCAGAAATCTTCACGTTTTTG |
| shcGAS, #2R | AATTCAAAAACGTGAAGATTTCTGCACCTAACTCGAGTTAGGTGCAGAAATCTTCACG |
| shSTING, #1F | CCGGGTTTACAGCAACAGCATCTATCTCGAGATAGATGCTGTTGCTGTAAACTTTTTG |
| shSTING, #1R | AATTCAAAAAGTTTACAGCAACAGCATCTATCTCGAGATAGATGCTGTTGCTGTAAAC |
| shSTING, #2F | CCGGGCATGGTCATATTACATCGGACTCGAGTCCGATGTAATATGACCATGCTTTTTG |
| shSTING, #2R | AATTCAAAAAGCATGGTCATATTACATCGGACTCGAGTCCGATGTAATATGACCATGC |

# **Table S3.** Antibodies.

| Protein | Resource | Number |
| --- | --- | --- |
| β-Actin | Beyotime | AA128 |
| DNMT1 | CST | 5032S |
| cGAS | CST | 15102S |
| cGAS (Mouse Specific) | CST | 31659S |
| STING | CST | 13647S |
| TRIF | CST | 4596S |
| RIG-I | CST | 4200S |
| MDA5 | CST | 5321S |
| MAVS | CST | 83000S |
| TBK1 | CST | 3504S |
| Phospho-TBK1 | CST | 5483S |
| IRF3 | CST | 11904S |
| IRF7 | CST | 39659S |
| STAT1 | CST | 14994S |
| Phospho-STAT1 | CST | 9167S |
| Phospho-Histone H2A.X | CST | 9718T |
| DNMT3A | CST | 49768T |
| DNMT3B | Absin | abs117756 |
| CD8 | CST | 98941S |
| CD3 | CST | 99940S |
| dsDNA antibody | Abcam | ab27156 |
| J2 | Scicons | 10010200 |
| Cy3-conjugated Affinipure Goat Anti-Mouse IgG (H+L) | Proteintech | SA00009-1 |
| FITC-conjugated Affinipure Goat Anti-Mouse IgG (H+L) | Proteintech | SA00003-2 |
| HRP-labeled Goat Anti-Rabbit IgG | Beyotime | A0208 |
| HRP-labeled Goat Anti-Mouse IgG | Beyotime | A0216 |

# **Table S4.** Expression scores of cGAS and STING, as well as CD8, PD-L1, and PD-1 positivity rates in breast cancer tissue array.

| **Location** | **cGAS**  **Score** | **STING**  **Score** | **CD8** | | **PD-L1** | | **PD-1** | |
| --- | --- | --- | --- | --- | --- | --- | --- | --- |
|  |  |  | **Positivy**  **rate** | **>10%**  **ratio** | **Positivy**  **rate** | **>10%**  **ratio** | **Positivy**  **rate** | **>10%**  **ratio** |
| A11 | 0 | 0 | 15% | **0.178** | 5% | **0.089** | 3% | **0.044** |
| D12 | 0 | 0 | 2% |  | < 1% |  | 0% |  |
| E07 | 0 | 0 | 5% |  | 5% |  | 1% |  |
| E08 | 0 | 0 | 2% |  | 5% |  | 1% |  |
| F01 | 0 | 0 | 30% |  | 50% |  | 20% |  |
| F02 | 0 | 0 | 8% |  | 1% |  | 1% |  |
| F09 | 0 | 0 | 5% |  | 2% |  | 2% |  |
| F12 | 0 | 0 | 3% |  | < 1% |  | < 1% |  |
| G02 | 0 | 0 | 5% |  | < 1% |  | < 1% |  |
| G03 | 0 | 0 | 2% |  | 1% |  | 0% |  |
| G04 | 0 | 0 | 3% |  | < 1% |  | 1% |  |
| A01 | 0 | 1 | 10% |  | < 1% |  | 1% |  |
| A05 | 0 | 1 | 3% |  | 10% |  | < 1% |  |
| C01 | 0 | 1 | 3% |  | 5% |  | 1% |  |
| C05 | 0 | 1 | 10% |  | 30% |  | 5% |  |
| C09 | 0 | 1 | 5% |  | < 1% |  | 1% |  |
| D04 | 0 | 1 | 10% |  | < 1% |  | 1% |  |
| D05 | 0 | 1 | 1% |  | < 1% |  | < 1% |  |
| D07 | 0 | 1 | 3% |  | 1% |  | < 1% |  |
| E03 | 0 | 1 | 2% |  | 0% |  | 0% |  |
| E10 | 0 | 1 | 5% |  | 5% |  | 1% |  |
| G01 | 0 | 1 | 10% |  | 7% |  | 3% |  |
| G08 | 0 | 1 | 5% |  | 0% |  | < 1% |  |
| G09 | 0 | 1 | 15% |  | 5% |  | 3% |  |
| G11 | 0 | 1 | 2% |  | 2% |  | < 1% |  |
| D03 | 1 | 0 | < 1% |  | 1% |  | 0% |  |
| D08 | 1 | 0 | 1% |  | 0% |  | 0% |  |
| E01 | 1 | 0 | 30% |  | 30% |  | 15% |  |
| G07 | 1 | 0 | 3% |  | 2% |  | < 1% |  |
| H01 | 1 | 0 | 8% |  | 2% |  | 3% |  |
| A03 | 1 | 1 | 1% |  | 10% |  | 0% |  |
| B01 | 1 | 1 | 30% |  | 5% |  | 3% |  |
| B03 | 1 | 1 | 5% |  | 10% |  | 1% |  |
| B05 | 1 | 1 | 5% |  | 1% |  | 0% |  |
| B11 | 1 | 1 | 10% |  | 5% |  | 1% |  |
| C11 | 1 | 1 | 5% |  | 0% |  | 0% |  |
| C12 | 1 | 1 | 10% |  | 1% |  | 1% |  |
| D01 | 1 | 1 | 20% |  | 30% |  | 7% |  |
| D06 | 1 | 1 | 10% |  | 5% |  | 2% |  |
| E04 | 1 | 1 | 15% |  | 0% |  | 5% |  |
| E09 | 1 | 1 | 20% |  | 1% |  | 8% |  |
| F03 | 1 | 1 | 3% |  | 10% |  | 2% |  |
| F11 | 1 | 1 | 10% |  | 5% |  | 3% |  |
| G05 | 1 | 1 | 10% |  | 1% |  | < 1% |  |
| G06 | 1 | 1 | 8% |  | 10% |  | 3% |  |
| C10 | 0 | 2 | 8% | **0.067** | 3% | **0.133** | < 1% | **0** |
| D10 | 0 | 2 | 3% |  | 1% |  | < 1% |  |
| E11 | 0 | 2 | 5% |  | 1% |  | 1% |  |
| F08 | 0 | 2 | 10% |  | 2% |  | 5% |  |
| G12 | 0 | 2 | 3% |  | 25% |  | 8% |  |
| B09 | 0 | 3 | 1% |  | 10% |  | < 1% |  |
| A09 | 1 | 2 | 7% |  | 1% |  | 3% |  |
| E02 | 1 | 2 | 2% |  | 0% |  | 0% |  |
| E06 | 1 | 2 | 7% |  | 1% |  | < 1% |  |
| D09 | 1 | 3 | 5% |  | 5% |  | 3% |  |
| A07 | 2 | 1 | 3% |  | 1% |  | < 1% |  |
| D02 | 2 | 1 | 5% |  | 1% |  | 1% |  |
| F04 | 2 | 1 | 5% |  | 1% |  | 1% |  |
| F10 | 2 | 1 | 20% |  | 25% |  | 7% |  |
| H02 | 3 | 1 | 3% |  | 2% |  | 1% |  |
| C03 | 2 | 2 | 7% | **0.6** | 10% | **0.5** | 5% | **0.2** |
| D11 | 2 | 2 | 7% |  | 5% |  | 5% |  |
| E05 | 2 | 2 | 30% |  | 30% |  | 15% |  |
| F05 | 2 | 2 | 3% |  | 5% |  | 1% |  |
| F06 | 2 | 2 | 15% |  | 10% |  | 10% |  |
| G10 | 2 | 2 | 35% |  | 5% |  | 10% |  |
| B07 | 2 | 3 | 20% |  | 30% |  | 1% |  |
| E12 | 2 | 3 | 5% |  | 50% |  | 1% |  |
| C07 | 3 | 2 | 35% |  | 35% |  | 25% |  |
| F07 | 3 | 2 | 15% |  | 45% |  | 10% |  |
