# Supplementary figures and images for: DNMT inhibition epigenetically restores the cGAS-STING pathway and activates RIG-I/MDA5-MAVS to enhance antitumor immunity
Source: Acta Pharmacol Sin. 2025 Aug 19;47(1):197–208. doi: 10.1038/s41401-025-01639-y (PMC12764874; doi:10.1038/s41401-025-01639-y)

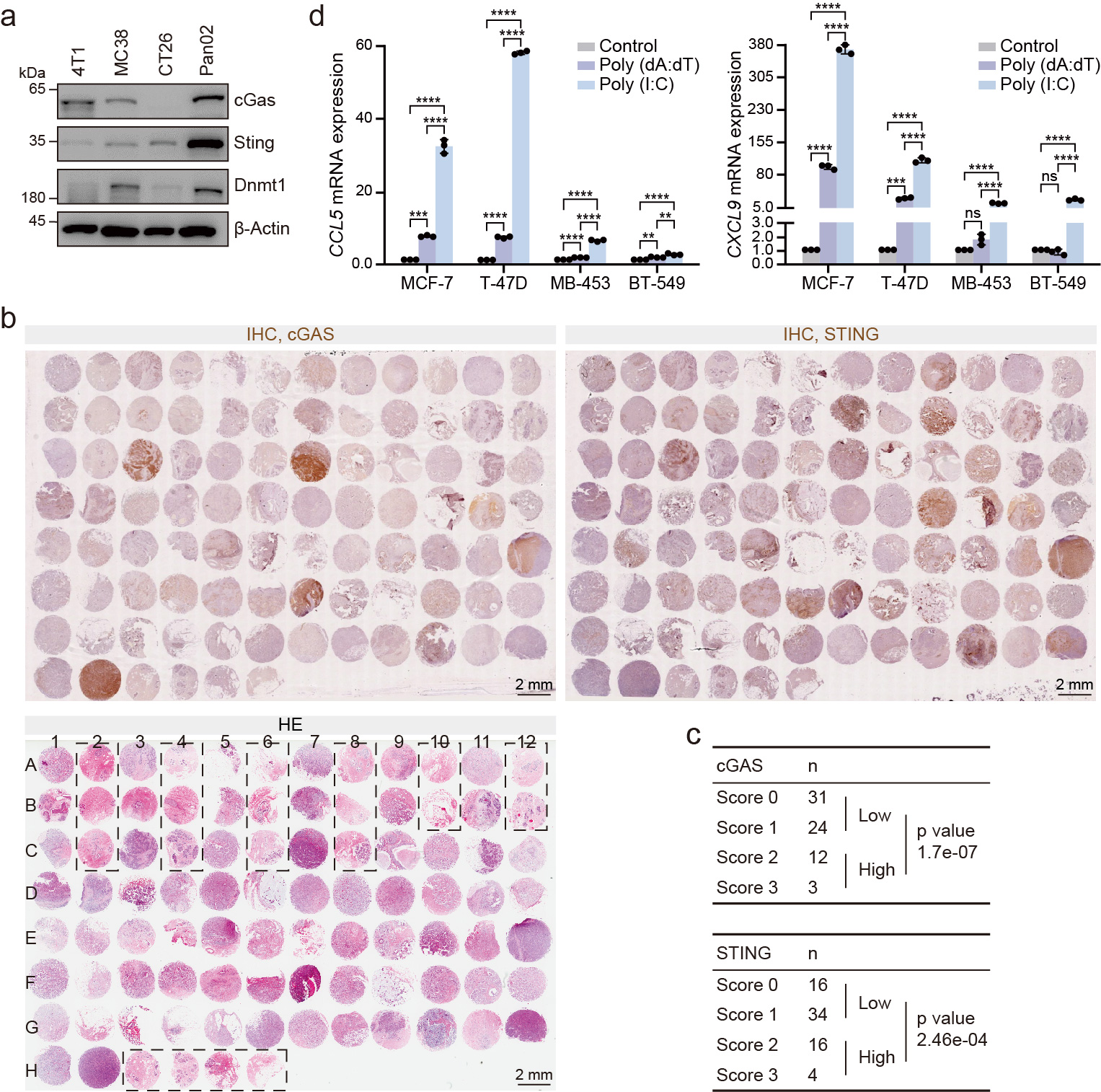

Supplement: Supplementary file 2 — Figure S1 [file 41401_2025_1639_MOESM2_ESM.jpg]

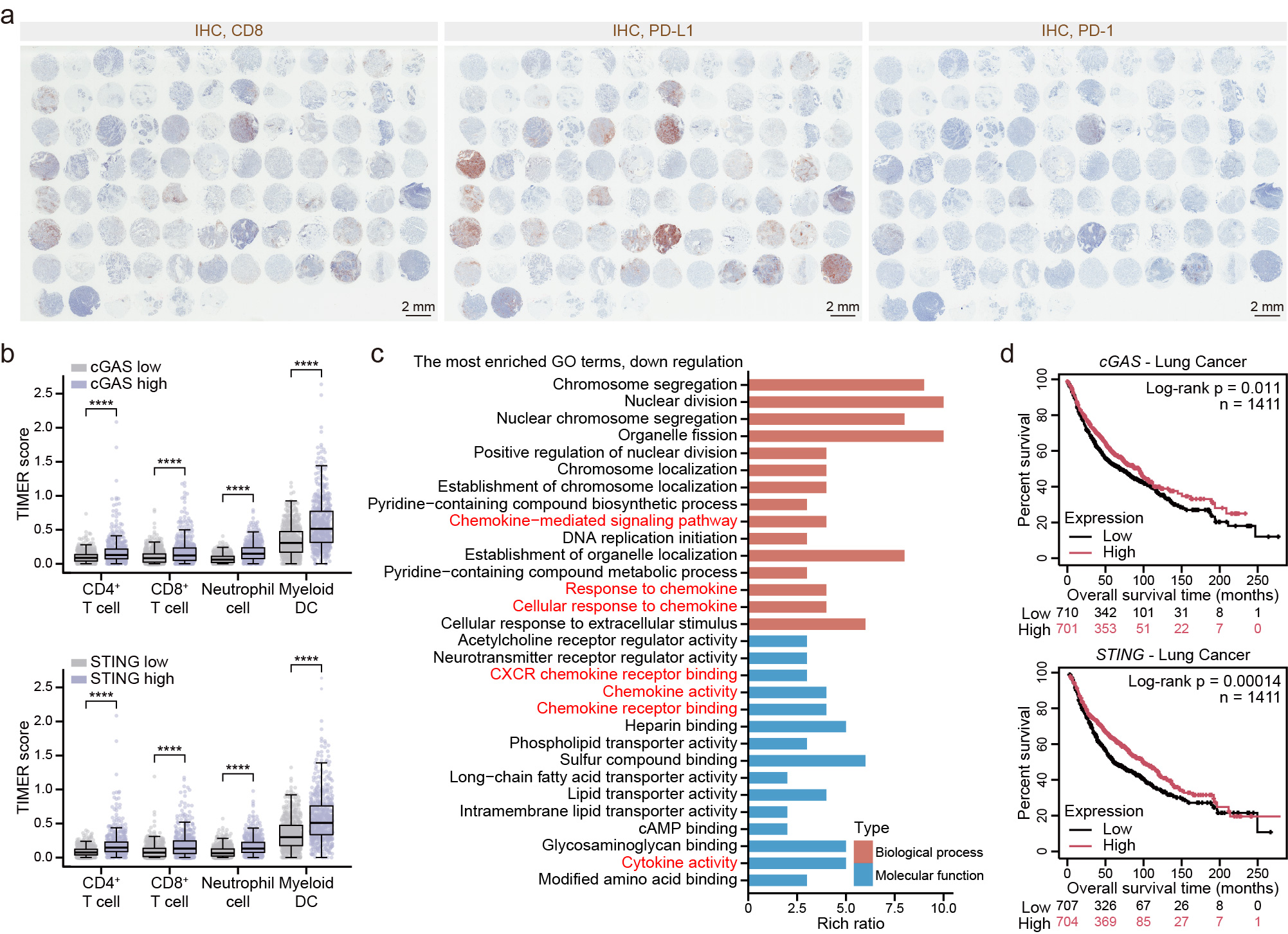

Supplement: Supplementary file 3 — Figure S2 [file 41401_2025_1639_MOESM3_ESM.jpg]

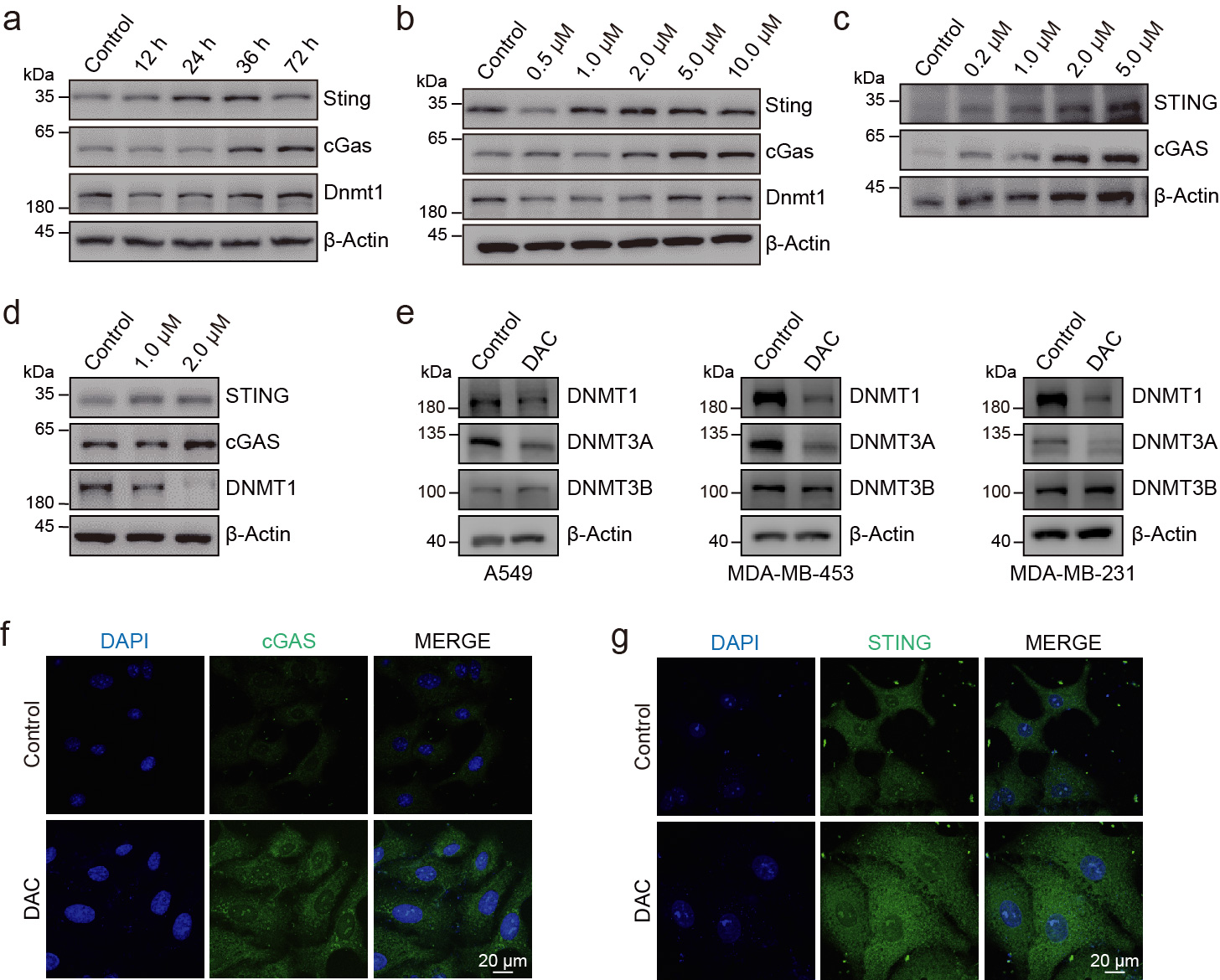

Supplement: Supplementary file 4 — Figure S3 [file 41401_2025_1639_MOESM4_ESM.jpg]

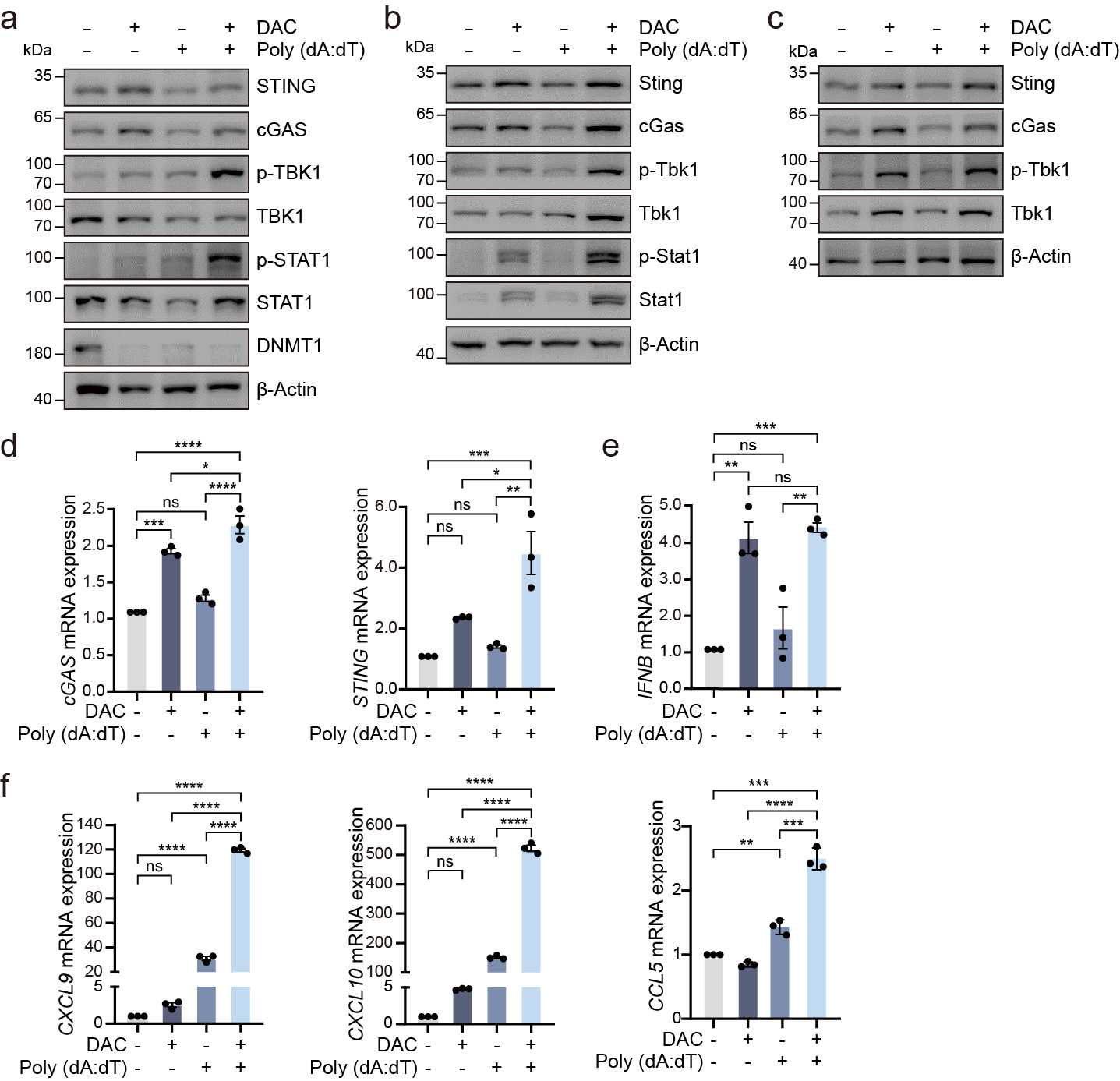

Supplement: Supplementary file 5 — Figure S4 [file 41401_2025_1639_MOESM5_ESM.jpg]

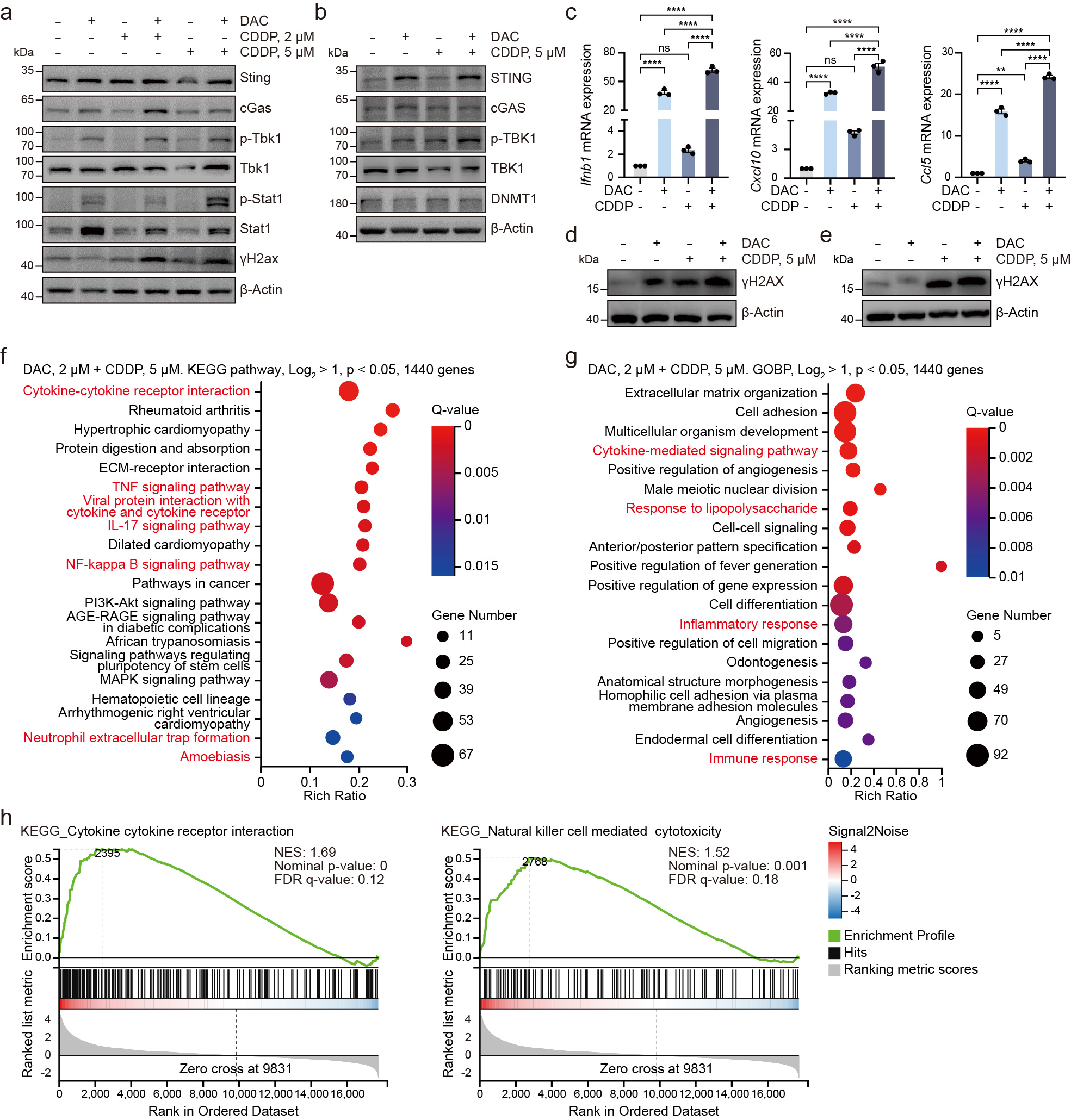

Supplement: Supplementary file 6 — Figure S5 [file 41401_2025_1639_MOESM6_ESM.jpg]

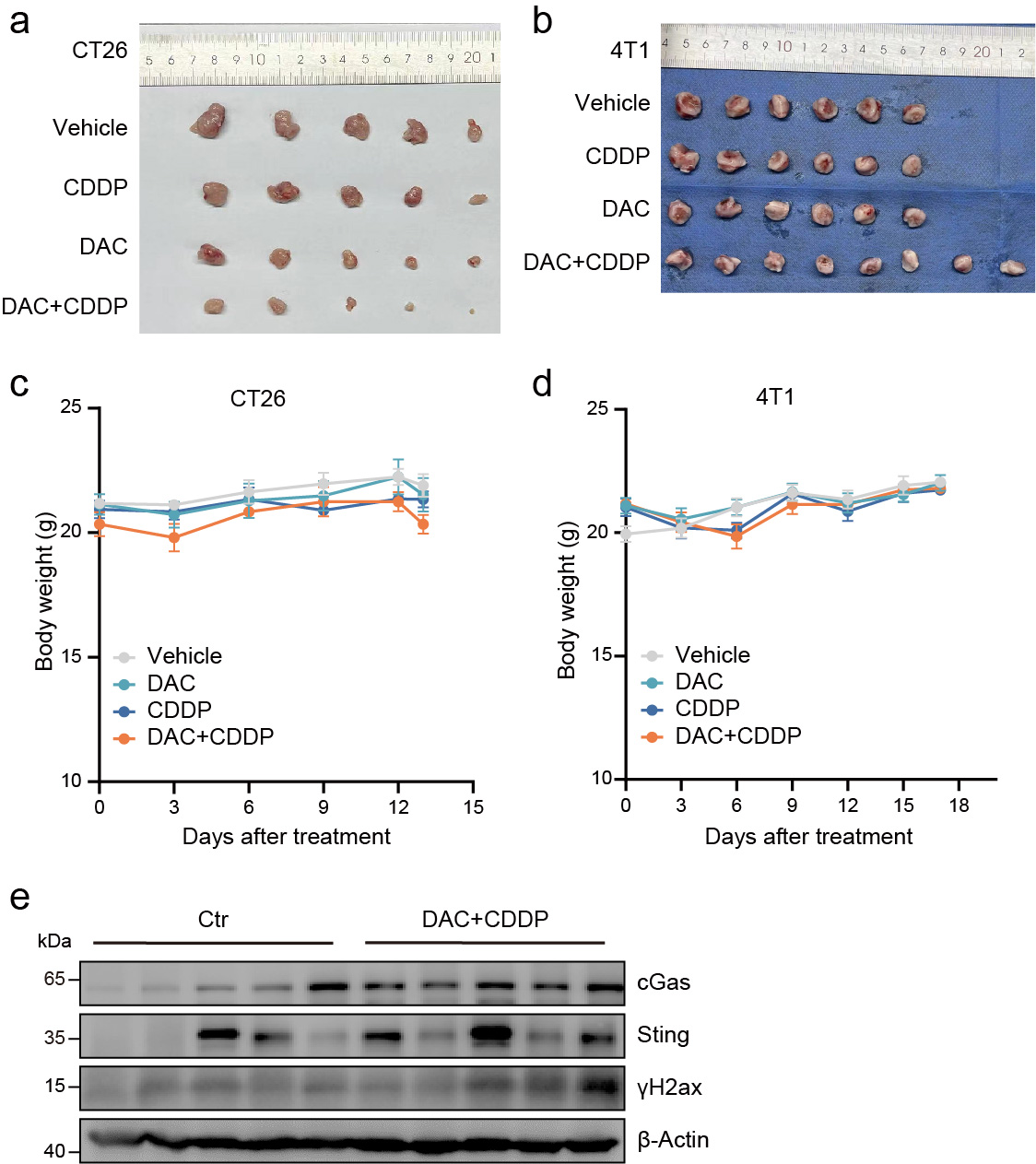

Supplement: Supplementary file 7 — Figure S6 [file 41401_2025_1639_MOESM7_ESM.jpg]
